# Genome-Wide Epigenetic Characterization of Tissues from Three Germ Layers Isolated from Sheep Fetuses

**DOI:** 10.3389/fgene.2017.00115

**Published:** 2017-09-04

**Authors:** Emanuele Capra, Paola Toschi, Marcello Del Corvo, Barbara Lazzari, Pier A. Scapolo, Pasqualino Loi, John L. Williams, Alessandra Stella, Paolo Ajmone-Marsan

**Affiliations:** ^1^Istituto di Biologia e Biotecnologia Agraria, Consiglio Nazionale delle Ricerche Lodi, Italy; ^2^Facoltà di Veterinaria, Università degli Studi di Teramo Teramo, Italy; ^3^Istituto di Zootecnica, Università Cattolica del Sacro Cuore Piacenza, Italy; ^4^Parco Tecnologico Padano Lodi, Italy; ^5^Davies Research Centre, School of Animal and Veterinary Sciences, University of Adelaide, Adelaide SA, Australia

**Keywords:** methylation, fetus, tissue, epigenomic, ectoderm, endoderm, mesoderm

## Abstract

DNA methylation of regulatory and growth-related genes contributes to fetal programming which is important for maintaining the correct development of three germ layers of the embryo that develope into different tissues and organs, and which persists into adult life. In this study, a preliminary epigenetic screen was performed to define genomic regions that are involved in fetal epigenome remodeling. Embryonic ectodermic tissues (origin of nervous tissue), mesenchymal tissues (origin of connective and muscular tissues), and foregut endoderm tissues (origin of epithelial tissue), from day 28 sheep fetuses were collected and the distribution of methylated CpGs was analyzed using whole-genome bisulfite sequencing. Patterns of methylation among the three tissues showed a high level of conservation of hypo-methylated CpG islands CGIs, and a consistent level of methylation in regulatory genetic elements. Analysis of tissue specific differentially methylated regions, revealed that 20% of the total CGIs differed between tissues. A proportion of the methylome was remodeled in gene bodies, 5′ UTRs and 3′ UTRs (7, 11, and 11%, respectively). Genes with overlapping differentially methylated regions in gene bodies and CGIs showed a significant enrichment for tissue morphogenesis and development pathways. The data presented here provides a “reference” for the epigenetic status of genes potentially involved in the maintenance and regulation of fetal developmental during early life, a period expected to be particularly prone to epigenetic alterations induced by environmental and nutritional stressors.

## Introduction

Methylation of CpG dinucleotides in the mammalian genome is a heritable epigenetic mark and also serves as an important mediator between the environment and genome function. Epigenetic modifications in regulatory and growth-related genes influence fetal development, with effects on important traits later in life (Bird, 2007). DNA methylation of cytosine residues, particularly at CpG dinucleotides, has been identified as an important regulatory mechanism of genome function, mediating genomic imprinting, embryo development (Razin and Shemer, 1995) and X-chromosome inactivation (Heard et al., 1997). CpG methylation may change through the course of development and aging (Madrigano et al., 2012), and in response to environmental conditions (e.g., nutrition; Murdoch et al., 2016) or disease (Bind et al., 2012; Leonard et al., 2012). Errors in methylation programming may cause developmental abnormalities and problems during pregnancy (Banister et al., 2011; Lambertini et al., 2011; Rexhaj et al., 2011; Jakovcevski and Akbarian, 2012). DNA methylation is mostly erased shortly after conception, followed by an increase in global DNA methylation around embryo implantation (Smith et al., 2012; Guo et al., 2014). During mammalian development the DNA methylome is then extensively remodeled in different cell lineages during differentiation. These dynamic changes lead to unique DNA methylation signatures in adult tissues, consistent with cell lineage and function (Reik et al., 2001; Bird, 2002; Smith and Meissner, 2013; Messerschmidt et al., 2014). Recent whole genome studies of humans (Yuen et al., 2011; Eckmann-Scholz et al., 2012; Slieker et al., 2015) and mice (Kremenskoy et al., 2003; Song et al., 2009) have identified tissue specific differentially methylated regions (tDMRs) during early development. The level of methylation of these regions then declines in adult tissues following an active demethylation wave during growth which continues gradually with aging.

In this work, variations in methylation among tissues during early mammalian development was assessed in all three germ layers of sheep fetuses by whole-genome bisulfite sequencing (WGBS).

The objective was to identify genomic regions with specific epigenetic signatures that differ between tissues. Sheep fetuses were used because of their similarity to human fetuses and potential for high external stressor exposure on farms. In particular we selected sheep since the developmental biology of the ovine fetus resembles closely the human situation displaying a comparable birth weight, organogenesis, and growth rate (Vonnahme et al., 2003; Wolfs et al., 2012). Moreover due to its long gestational period and tolerance for intrauterine surgery, among livestock species, the pregnant sheep is routinely used to investigate fetal programming, fetal growth, metabolism, and nutrition (Gopalakrishnan et al., 2004; McMillen and Robinson, 2005; Sinclair et al., 2007; Hyatt et al., 2008). For obvious ethical reason previous works describing tissue specific DMR on humans have been done in adult tissues (Lokk et al., 2014) or in fetal tissues collected from abortion material (Slieker et al., 2015), therefore important information about dynamics of DMR changes during normal development are still needed. In addition, these data provide a reference to investigate epigenetic changes induced by, e.g., environmental or nutritional stress during gestation (Waterland and Michels, 2007; Gluckman et al., 2009) which has not been investigated in fetal tissues of livestock species. The influence of external factors such as nutritional status during development that lead to epigenetic modifications and subsequently phenotypic variation, may in part be responsible for discrepancies between predicted breeding values and observed performance and is hence of interest for improving animal breeding and management (Murdoch et al., 2016).

## Materials and Methods

### Animal Treatment and Tissue Collection

Sardinian ewes obtained from local breeders were housed at the Istituto Zooprofilattico Abruzzo (Loc. Gattia, Italy) authorized experimental farm. The ewes were fed and kept under the best sheep housing standards. All animal experiments were performed in accordance with Italian animal experimentation legislation (DPR 27/1/1992 Animal Protection Regulations of Italy) in concordance with European Community regulation 86/609 and were ethically approved by CEISA (Inter-Institutional Ethics Committee for Animal Experimentation Prot. 79/2013/CEISA Prog. 58. The permit no.: CEISA VI, Classe 8.1, Prot. 2823).

**FIGURE 1:**
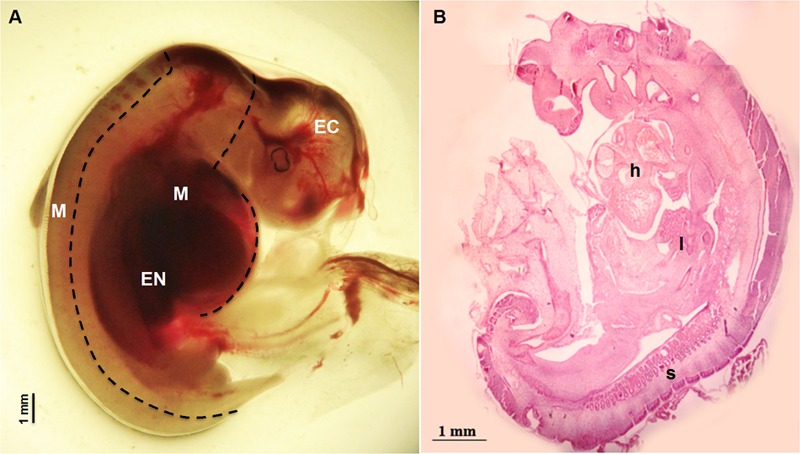
Fetus at 28 day of gestation: **(A)** bright field image of a fetus with the dashed line illustrating the dissection lines for tissue sampling: EC indicates Ectodermal tissue (cephalic structures); M indicates Mesodermal tissue (somites and heart) and EN indicates foregut Endodermal tissue (liver); **(B)** histological view showing the distribution of the developing tissues: h denotes the position of the heart position, l the developing site of the liver and s visible somites.

Sheep were synchronized for ovulation with 25 mg Crono-gests sponges (Intervet, Milan, Italy) and naturally mated. Fetal measurement and tissues collection: three fetuses were surgically collected at 28 day of gestation by paramedian laparatomy under general anesthesia. Conceptuses were immediately immersed in pre-warmed phosphate-buffered saline (PBS) and examined under a stereomicroscope to verify their vitality by the presence of a heartbeat. For each fetus, crown-rump measurement was recorded in triplicate using Image J software, an open source image-processing program designed for scientific multidimensional images. Fetuses were transferred to clean Petri dishes containing ice cold PBS and the Amniotic sac was removed to expose the fetus. Tissues were collected under a dissection microscope using forceps to stabilize the fetus and micro-scissors or a blade to isolate the three germ layer tissues (**Figure 1**): Ectodermal tissue EC (cephalic structures); Mesodermal tissue M (somites and heart); foregut Endodermal tissue EN (liver). Samples were snap frozen in liquid nitrogen and stored at -80°C for subsequent analysis. Examples of fetal tissues were fixed in 4% paraformaldehyde, embedded in Paraplast and hematoxylin-and-eosin and stained as described by Fidanza et al. (2014). Pictures were taken using the Nikon Eclipse E600 microscope.

### DNA Isolation, Library Preparation, and Sequencing

Genomic DNA from each tissue was isolated using the NucleoSpin^®^ Tissue kit (Macherey-Nagel, Düren, Germany), following the manufacturer’s instructions. DNA concentration was estimated by PicoGreen^®^ (Thermo Fisher Scientific, Waltham, MA, United States). Libraries were generated using the TruSeq^®^ DNA PCR-Free Library Preparation Kit (Illumina) including a step of bisulfite treatment. After ligation of adapters, samples were converted with EpiTect Bisulfite Kits (Qiagen) and finally PCR amplified with KAPA HiFi Uracil+ (Kapa Biosystems). WGBS was performed on an Illumina Hiseq 2000 (San Diego, CA, United States) to generate 100-base paired-end reads.

### Bioinformatic Analysis

Preliminary quality control of raw reads was carried out with FastQC^1^. Illumina raw sequences were then filtered with Trimmomatic software to remove adapters and low quality bases at the ends of sequence, using a sliding window approach. Data are available in the Sequence Reads Archive (SRA), BioProject accession number, PRJNA385562.

Bismark software v.0.17.0 was used to align each read to a bisulfite-converted sheep genome (Oar_v3.1) with option -N 1, and methylation calls were extracted using *Bismarkmethylation_extractor* function. Seqmonk software (version 0.34.1)^2^ was used for visualization and analysis of the Bismark output. Methylated regions (MRs) were detected by dividing the genome in 100 bp tiles and analyzing average methylation for tiles containing at least four mutually covered Cs in CpG context per position. Only regions sequenced in all tissues for at least two samples were retained to investigate enrichment for different features, 5′ UTR, 3′ UTR and gene bodies, in CpG islands, shores and shelves. 5′ and 3′ UTRs were defined as 2000 bp upstream or downstream of a gene. Differentially methylated regions (DMRs) were calculated for each tissue (*n* = 3) vs. the others two tissues (*n* = 4–6; at least two samples for both tissues) using the logistic regression filter in R to assess differential methylation (FDR ≤ 0.05, absolute cut-off of 5%). Hierarchical clustering was calculated for DMRs present in CGIs, genes (FDR < 10exp-7), 5′ UTRs and 3′ UTRs. The level of methylation was normalized between samples and methylation percentage from a selection of DMRs showing the highest differences in methylation was used for clustering using the Genesis software (Sturn et al., 2002).

A list of DMRs found for at least one tissue vs. the other two tissues (DMRs EC + DMRs M + DMRs EN) for CGIs and different genomic features was created for pathway analysis. Gene ontology (GO) classification of the DMRs was performed according to classical GO categories, using the Cytoscape plug-in ClueGO which integrates GO (Bindea et al., 2009) and enhances biological interpretation of large lists of genes.

## Results

### Fetal Tissue Isolation

Fetuses collected at 28 day of gestation were measured and crown-rump length was 12.60 ± 0.179 mm (Mean ± SEM) (**Figure 1**).

### Sequencing Statistic and CpG Distribution

The average number of reads per sample was 38.7 M (ranging from 25.2 to 77.3 M) with a high mapping efficiency on the sheep reference genome for all samples (range between 75.6 and 79.8%). After calculating cytosine methylation conversion rate, the sequencing coverage for each cytosine was estimated to be about 2.5X (see Supplementary Table S1 for statistics). After applying a more stringent cut off of 4X methylated cytosine coverage, a total of 25.8 M methylated region (MR) of 100 bp were identified using SeqMonk software which spanned all the sheep genome. Among these, a total of 163,209 MRs, observed in at least one of the three tissues (*n* = 3) and at least two in the other tissues (*n* = 4–6) were selected to compare the DNA cytosine methylation profile.

**FIGURE 2:**
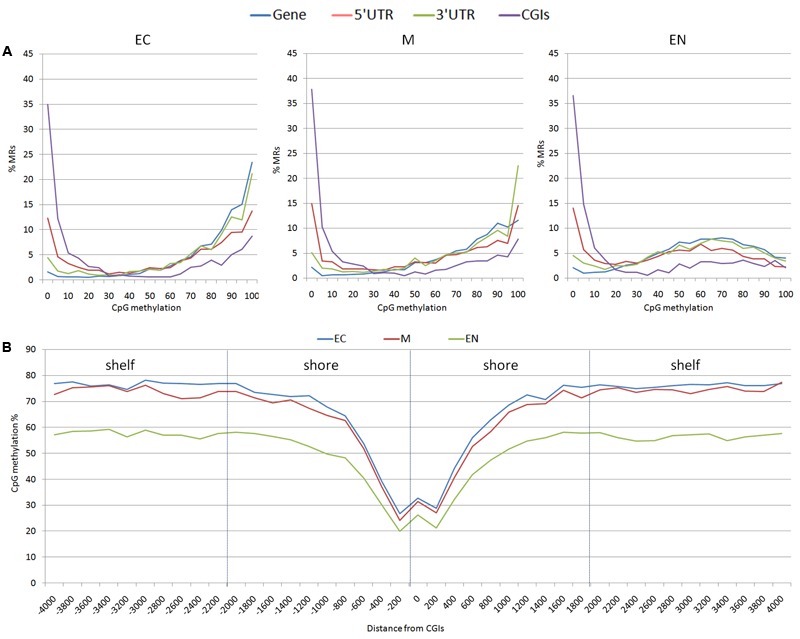
Distribution of methylation sites: **(A)** methylated regions (MRs) were stratified based on the average methylation level of CpGs (ranging from 0 to 100%), **(B)** CpG methylation percentage for each position surrounding the CGIs; shores (±2 Kb of CGIs) and shelves (±2 Kb of shores) were shown. EC, M, and EN indicate Ectodermal, Mesodermal, and Endodermal tissues respectively.

From the 163,209 MRs, a total of 63,290 MRs were located in 10,571 out of the 24,142 mapped genes. There were 4,835 MRs upstream (-2 Kb) and 2,942 downstream (+2 Kb) of genes, and 1,459 were located within the 19,450 CpG islands (CGIs) mapped. Considering CpG methylation frequency, genes and 3′ UTR of genes were prevalently hyper-methylated, whereas 5′ UTR, and CGIs showed a higher proportion of hypo-methylated regions (**Figure 2A**). The CpG methylation level in the CpG island shores (±2 Kb of CGIs; 7,987 MRs) and shelves (±2 Kb of shores; 5,806 MR) increased moving away from CGIs. A lower level of methylation was observed in the EC tissue (**Figure 2B**). CpG methylation at 1,459 MRs located in CGIs was examined in the three tissues: EC had 870 MRs, M 868 MRs and EN 915 MRs with low CpG methylation status (defined as a region with DNA methylation 0 ≤ α ≤ 0.2). Intriguingly, 797 low methylated regions (about 84.4%) were shared among the three tissues, whereas hypermethylated CGIs (0.8 ≤ α ≤ 1.0) were less conserved (about 32.0%) (Supplementary Figure S1). Transcription start sites (TSSs) also differed in hypomethylated versus hypermethylated CGIs regions: hypo-methylated regions were enriched in TSS elements and clearly positioned within a few 100 bp of TSSs, whereas TSSs were poor in hyper-methylated CGI and distributed over more distal positions (Supplementary Table S2 and Figure S2).

### Differentially Methylated Region in the Three Tissues

**FIGURE 3:**
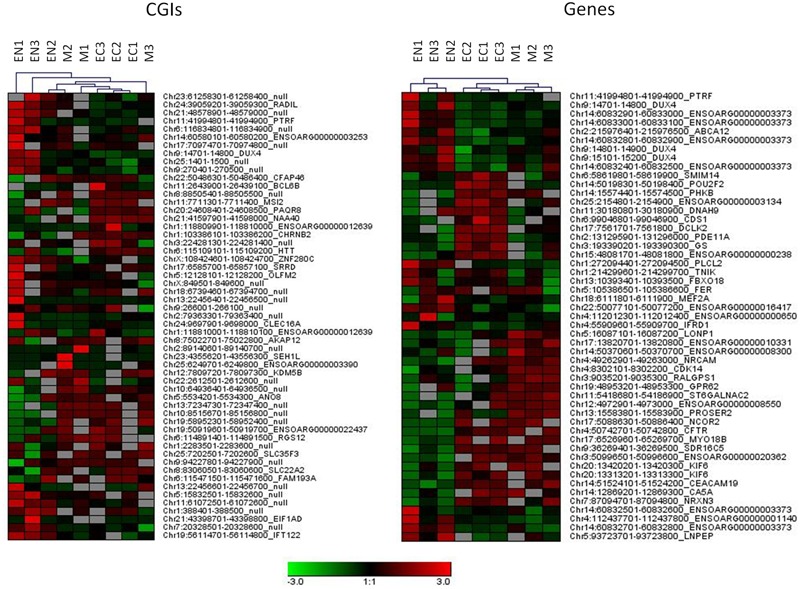
Hierarchical clustering for DMRs found in CGIs and gene bodies (DMRs, FDR < 10exp-7). Each tissue was compared with other two tissues and 20 more hyper and 20 more hypo methylated DMRs from each comparison were used for clustering samples.

A relatively large percentage of CGIs (20.2% of total) were differentially methylated across the three fetal tissues. Many of these (28.8%) were located near (±2 Kb) the TSSs and more than 46.1% were within well-characterized genes. A genome-wide analysis including genes and regulatory elements revealed that variation in CpG methylation occurred within genes, 5′ UTRs and 3′ UTRs regions (7.2, 11.1, and 10.9% of DMR/MRs) respectively (Supplementary Table S3 and **Data Sheet 4**). Hierarchical analysis of the most significant DMRs found in CGIs, in gene bodies, 5′ UTR and 3′ UTR discriminated among samples from different tissues (**Figure 3** and Supplementary Figure S3).

### Pathway Analysis

**FIGURE 4:**
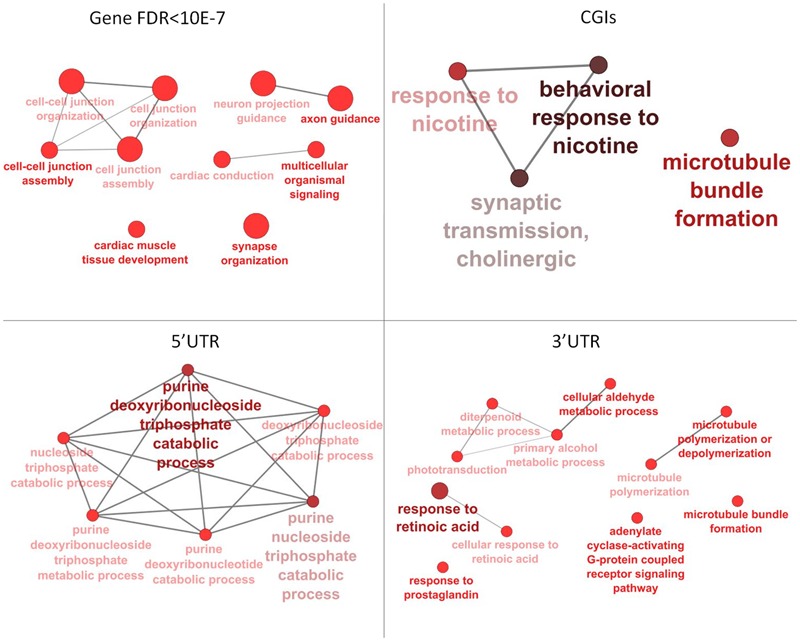
Pathway analysis for differentially methylated genes (DMGs) found in gene bodies, 5′ and 3′ UTR and CGIs. Pathway enrichment for DMGs found in gene bodies was calculated using only a subset of the most significant differentially methylated regions (DMRs) (FDR < 10-7).

Annotation of 4,554 DMRs that overlapped gene bodies led to 2,695 differentially methylated genes (DMGs). 535; 321 and 295 DMRs located near 5′ UTR, 3′ UTRs and CGIs, were close (±2 Kb) to 407; 284 and 90 DMGs respectively. Pathway analysis was performed on DMGs found in 5′ UTR, 3′ UTRs and CGIs, and a selection of DMGs (DMRs, *n* = 451, FDR < 10exp-7, overlapping 337 genes) in gene bodies (**Figure 4**). Variation in CpG methylation in gene bodies was predominantly found in pathways that were related to tissue morphogenesis and development such as multicellular organism signaling, cell–cell junction assembly and organization, cardiac muscle development and axon guidance and synapse organization. CpG methylation in CGIs occurred most frequently in genes related to nervous system regulation as shown in **Table 1**. Pathway analysis of DMRs in CGIs and gene bodies identified several related genes (members of the same gene family), or subunits of the same protein. These genes mapped at distant chromosomal locations (Supplementary Table S4).

**Table 1:** Pathways identified for the differentially methylated genes DMGs found between tissue in gene bodies (GENEs) and CpG islands (CGIs).

GO-ID	GO-term	Associated genes found	*p*-Value^∗^
**GENEs**			
GO:0035637	Multicellular organismal signaling	[AKAP9, CACNA1D, DSPP, GRIK2, HCN4, MEF2A, NFASC, NRCAM, PRKCA]	0.012387
GO:0007043	Cell–cell junction assembly	[APC, HEG1, INADL, MPP5, NEDD4L, NFASC, PRKCA, TRPV4]	0.012396
GO:0007411	Axon guidance	[CACNA1D, CD72, CLASP2, CNTN4, COL4A2, EPHA5, FZD3, ISPD, LAMA2, MATN2, MYCBP2, MYH11, NFASC, NRCAM, NRXN3, NTN1, SPTB, TRPC7]	0.014183
GO:0097485	Neuron projection guidance	[CACNA1D, CD72, CLASP2, CNTN4, COL4A2, EPHA5, FZD3, ISPD, LAMA2, MATN2, MYCBP2, MYH11, NFASC, NRCAM, NRXN3, NTN1, SPTB, TRPC7]	0.014183
GO:0050808	Synapse organization	[CHAT, CHRNB2, EPHA5, FARP1, FGFR2, NFASC, NRCAM, NRXN2, NRXN3, PDZRN3, WNT7A]	0.016662
GO:0034330	Cell junction organization	[APC, CDH12, CDH13, DLEC1, DSPP, HEG1, INADL, LAMA3, MPP5, NEDD4L, NFASC, PRKCA, TRPV4]	0.01855
GO:0045216	Cell–cell junction organization	[APC, CDH12, CDH13, DLEC1, DSPP, HEG1, INADL, MPP5, NEDD4L, NFASC, PRKCA, TRPV4]	0.019087
GO:0034329	Cell junction assembly	[APC, CDH12, CDH13, DLEC1, HEG1, INADL, LAMA3, MPP5, NEDD4L, NFASC, PRKCA, TRPV4]	0.023111
GO:0061337	Cardiac conduction	[AKAP9, CACNA1D, DSPP, HCN4, MEF2A, PRKCA]	0.041008
GO:0048738	Cardiac muscle tissue development	[DSPP, ERBB4, FGFR2, FHOD3, HEG1, MEF2A, MYH11, MYO18B, SOX6, ZFPM2]	0,046303
**CGIs**			
GO:0035095	Behavioral response to nicotine	[CHRNA4, CHRNB2, CHRNB4]	9.14E-06
GO:0007271	Synaptic transmission, cholinergic	[ACHE, CHRNA4, CHRNB2, CHRNB4]	1.97E-05
GO:0035094	Response to nicotine	[CHRNA4, CHRNB2, CHRNB4]	0.001767
GO:0001578	Microtubule bundle formation	[CFAP46, DNAAF5, NAV1]	0.002852

*Indicates are gene ontology IDs (GO-ID), gene ontology terms (GO-term), associated genes found and corrected *p*-values as determined by ClueGO (http://apps.cytoscape.org/apps/cluego).*

*^∗^Corrected with Bonferroni.*

## Discussion

In this work 4X coverage WGBS profiling was used to characterized patterns of CpG methylation in three different tissues from sheep fetuses. This coverage is more than the 2.5–3X which has been shown to be sufficient for the identification of DMRs with large methylation differences (Ziller et al., 2015).

Distribution of methylated sites observed across different gene features was similar to the DNA methylation landscape reported for human fetal tissue obtained with a 450 k methylation array (Huse et al., 2015; Slieker et al., 2015). CGIs across the genome have been found to have low DNA methylation in fetal tissues, while the level of CpG methylation is higher in shores and shelves moving away from CGIs, which is consistent with the stably methylated sequences surrounding CpG islands reported by Edgar et al. (2014).

Hypo-methylated CGIs become established during the early phase of cell lineage differentiation, and appears to be highly stable across diverse developmental states and cell types (Straussman et al., 2009). This was also observed in the present study with more than 84% hypo-methylated CGIs shared among fetal tissues which tended to be near TSSs. TSSs hypomethylation is likely established during early development and TSSs have been proposed as potential regulatory domains that mediate transcription (Deaton and Bird, 2011).

In contrast to the relatively subtle changes in hypomethylated CGIs, only a small proportion of methylome (32%) with higher level of methylation in CGIs was conserved among the three tissues studied. About 20% of the CGIs were differentially methylated among the three tissues. In a study of nearly 800 genes, Yuen et al. (2011), found that 23% of these were differentially methylated between different somatic tissues.

Each of the tissues examinated in the present study was composed of several cell types and the observed methylation profile was therefore an average. Assessment of ELISA-based global DNA methylation quantification in fetal tissues at the first trimester showed variations in the methylation level in the different tissues (Fa et al., 2016). In the present work, the endoderm had a lower overall level methylation whereas mesoderm showed fever DMRs.

Although tissue-specific methylation of CGIs was observed at many loci that are essential for development including HOX and PAX family members (Illingworth et al., 2008), it has also been shown that gene-body DNA methylation plays a role in tissue-specific gene regulation (Yagi et al., 2008; Wan et al., 2015).

Tissue-specific methylation was observed for both CpG in CGIs and CpG in gene bodies outwith CGIs. GO analysis for DMRs located in gene bodies and CGIs showed an overall enrichment of genes involved in tissue morphogenesis, regulation and development. DMRs in CGIs overlapped many genes belonging to cholinergic signaling, including *ache, chrnA4, chrnB2, chrnB4*. This pathway seems to be involved in nervous system development and behavior: analysis of methylation variation in dorsolateral prefrontal cortex from individuals ranging from fetal to 84 years old, showed that methyl-CpG levels of *chrnB4* change over the human life span (Torabi Moghadam et al., 2016). Mice null for *chrnA4*, display an increased anxiety (Ross et al., 2000), and variation in CpG methylation of *chrnA4* in ventral-hippocampal granule cells and neurons is also linked to anxiety (Oh et al., 2013). The nicotine receptor subunits are crucial for the correct nervous system development in the brain of rat fetuses. Previous studies suggested that prenatal exposure to nicotine significantly increased mRNA expression of brain nicotine receptor subunits α2, α4, α7, and β2 units and is associated with abnormal development in fetuses, including fetal brain damage (Lv et al., 2008).

Comparison between fetal tissue in the present study revealed variation of CpG methylation in the gene bodies of several cell adhesion proteins, including; *ephA5; NRXN2, 3, wnt7A, cgh12, 13*, and *FZD3.* Maternal environment induces offspring methyl-CpG variation in related genes including *ephA4, A8, B1,B2*; *NRXN1, 2; wnt2, 2b, 3, 7a, 7b, 10b, 11; cdh 9, 11, 13*, and *FZD7* (Oh et al., 2013). Adhesion proteins play an essential role during fetal development. The variations in the methylation status of cell adhesion protein genes in placenta have been associated to pre-eclampsia, a condition during pregnancy causing a sudden rise in blood pressure and signs of damage to other organ systems, especially the kidney (Anton et al., 2014). CpGs methylation and mRNA expression in genes associated to Wnt signaling (*wnt* family) and neural development such as *nrxn2*, has been shown to be sensitive to environmental condition such as prenatal exposure to infectious or inflammatory insults (Richetto et al., 2017).

In the present study differences in CpG methylation were also found in genes related to cardiac muscle tissue development; *MYO18B, MEF2A, MYH11.* Deficiency of MYO18B in mice results in disruption of myofibrillar structures in embryonic cardiac myocytes (Ajima et al., 2008) while *MEF2A* deficiency is associated with myofibrillar disarray in embryonic heart development (Naya et al., 2002). However, no data are available linking methylation of *MYP18B* and *MEF2A* with expression in fetal tissues. Hypomethylation of *MYH11* has been observed in muscle tissue from pigs that have undergone constant heat stress and were affected in muscle development (Hao et al., 2016), although *MYH11* expression seems to be regulated by chromatin remodeling of histone acetylation rather than methylation status (Gomez et al., 2015).

## Conclusion

The WGBS profiling of ovine fetal tissues presented here provides a catalog of DNA methylation patterns across the sheep genome related to three developmental layers of the embryo. Many DMRs were layer specific and enriched in genes often closely related to specific developmental functions. Knowledge of the epigenetic status of genes potentially involved in the maintenance and regulation of normal developmental and differentiation provides a starting reference point for studies on the effects of environmental and nutritional stressors on development.

## Author Contributions

PL, JW, PA-M, and AS conceived the study and revised the manuscript. PT and PS isolated fetal tissue. EC performed DNA extraction, libraries preparation and sequencing. MDC and BL carried out the bioinformatic analysis. EC and PT wrote the manuscript. All authors read and approved the final manuscript.

## Conflict of Interest Statement

The authors declare that the research was conducted in the absence of any commercial or financial relationships that could be construed as a potential conflict of interest.

## Footnotes

1 http://www.bioinformatics.babraham.ac.uk/projects/fastqc/

2 http://www.bioinformatics.babraham.ac.uk/projects/seqmonk/

## Supplementary Material

DATA SHEET 4 Differentially methylated regions (DMRS) for the three germ layers tissues (EC; M; EN) found in different genomic regions: CGIs, genes, 5′ UTRs and 3′ UTRs. Each tissue was compared with other two tissues. For each DMR, Probe position, Feature, Orientation, Distance and FDR were reported.
