# Supplementary material for: Genome-Wide Epigenetic Characterization of Tissues from Three Germ Layers Isolated from Sheep Fetuses
Source: Front Genet. 2017 Sep 4;8:115. doi: 10.3389/fgene.2017.00115 (PMC5591608; doi:10.3389/fgene.2017.00115)
Supplement: Supplementary file 1 [file Table_1.DOCX]

| DataStore | Sequence pairs total | Mapping efficiency | methylated C's in CpG context total | Cytosine Fold Coverage |
| --- | --- | --- | --- | --- |
| HFTa | 25836840 | 79.3% | 76.9% | 0.017 |
| HFTb | 25243494 | 79.5% | 78.8% | 0.016 |
| HFTc | 36476402 | 75.6% | 74.7% | 0.025 |
| MFTa | 26105546 | 78.9% | 73.6% | 0.016 |
| MFTb | 27561319 | 77.8% | 75.3% | 0.019 |
| MFTc | 25789117 | 77.4% | 69.9% | 0.017 |
| LFTa | 77341515 | 78.3% | 46.8% | 0.049 |
| LFTb | 47687149 | 79.8% | 65.6% | 0.03 |
| LFTc | 56895518 | 77.0% | 52.6% | 0.033 |

**Supplementary Table S1.** Sequencing statistic of three biological replicates for EC, M, EN tissues
