# Supplementary material for: Genome-Wide Epigenetic Characterization of Tissues from Three Germ Layers Isolated from Sheep Fetuses
Source: Front Genet. 2017 Sep 4;8:115. doi: 10.3389/fgene.2017.00115 (PMC5591608; doi:10.3389/fgene.2017.00115)
Supplement: Supplementary file 2 [file Table_2.docx]

|  | 0≤α≤1.0 | 0≤α≤0.2 | 0.8≤α≤1 |
| --- | --- | --- | --- |
| CGIs | 1459 | 914 | 288 |
| CGIs near TSSs | 637 | 519 | 55 |
| CGIs/CGIs near TSSs % | 43.67 | 56.78 | 19.10 |

**Supplementary Table S2.** Number of transcription start sites TSSs found in CpG islands: total (0≤α≤1.0), hypomethylated (0≤α≤0.2) and hypermethylated (0.8≤α≤1) regions found at least in 1 tissue (n=3).
