# Supplementary material for: Genome-Wide Epigenetic Characterization of Tissues from Three Germ Layers Isolated from Sheep Fetuses
Source: Front Genet. 2017 Sep 4;8:115. doi: 10.3389/fgene.2017.00115 (PMC5591608; doi:10.3389/fgene.2017.00115)
Supplement: Supplementary file 3 [file Table_3.docx]

|  | CGI | Gene | 5'UTR | 3'UTR |
| --- | --- | --- | --- | --- |
| MRs | 1459 | 63290 | 4835 | 2942 |
| DMRs in EC | 176 | 1670 | 217 | 115 |
| DMRs in M | 109 | 1127 | 126 | 84 |
| DMRs in EN | 198 | 3176 | 362 | 214 |
| DMRs in all tissues | 295 | 4554 | 535 | 321 |
| EC DMRS/MRs % | 12.1 | 2.6 | 4.5 | 3.9 |
| M DMRS/MRs % | 7.5 | 1.8 | 2.6 | 2.9 |
| EN DMRS/MRs % | 13.6 | 5.0 | 7.5 | 7.3 |
| DMRs all tissues/MRS % | 20.2 | 7.2 | 11.1 | 10.9 |

**Supplementary Table S3.** Number of differential methylated regions DMRs found in EC, M, EN and in all tissue. The ratios between DMRs and methylated region MRs are reported.
