# Supplementary material for: Genome-Wide Epigenetic Characterization of Tissues from Three Germ Layers Isolated from Sheep Fetuses
Source: Front Genet. 2017 Sep 4;8:115. doi: 10.3389/fgene.2017.00115 (PMC5591608; doi:10.3389/fgene.2017.00115)
Supplement: Supplementary file 4 [file Table_4.DOCX]

| GENEs | | | | | | | | |  |
| --- | --- | --- | --- | --- | --- | --- | --- | --- | --- |
| GENE | | Description | | Chromosome | | start | | end |  |
| AKAP9 | A-kinase anchoring protein 9 | | [4](http://www.ensembl.org/Ovis_aries/Location/View?db=core;g=ENSOARG00000017010;r=4:9183142-9351553;t=ENSOART00000018537) | | 9183142 | | 9351553 | | |
| APC | adenomatous polyposis coli | | 7 | | 891318 | | 969935 | | |
| CACNA1D | calcium channel voltage-dependent L type alpha 1D subunit | | 19 | | 47153174 | | 47501739 | | |
| CD72 | CD72 molecule | | 2 | | 52594675 | | 52607206 | | |
| CDH12 | cadherin 12 | | [16](http://www.ensembl.org/Ovis_aries/Location/View?db=core;g=ENSOARG00000012806;r=16:51055562-51428550;t=ENSOART00000013916) | | 51055562 | | 51428550 | | |
| CDH13 | cadherin 13 | | [14](http://www.ensembl.org/Ovis_aries/Location/View?db=core;g=ENSOARG00000009566;r=14:8805141-9619968;t=ENSOART00000010416) | | 8805141 | | 9619968 | | |
| CHAT | choline O-acetyltransferase | | 25 | | 43333483 | | 43382252 | | |
| CHRNB2 | cholinergic receptor nicotinic beta 2 (neuronal) | | 1 | | 103382196 | | 103390463 | | |
| CLASP2 | cytoplasmic linker associated protein 2 | | [19](http://www.ensembl.org/Ovis_aries/Location/View?db=core;g=ENSOARG00000016028;r=19:7671705-7840908;t=ENSOART00000017468) | | 7671705 | | 7840908 | | |
| CNTN4 | contactin 4 | | 19 | | 23126508 | | 23921247 | | |
| COL4A2 | collagen type IV alpha 2 chain | | [10](http://www.ensembl.org/Ovis_aries/Location/View?db=core;g=ENSOARG00000006515;r=10:84344246-84506524;t=ENSOART00000007098) | | 84344246 | | 84506524 | | |
| DLEC1 | deleted in lung and esophageal cancer 1 | | [19](http://www.ensembl.org/Ovis_aries/Location/View?db=core;g=ENSOARG00000000853;r=19:11431733-11518468;t=ENSOART00000000917) | | 11431733 | | 11518468 | | |
| DSPP | dentin sialophosphoprotein | | 6 | | 102110796 | | 102116644 | | |
| EPHA5 | EPH receptor A5 | | [6](http://www.ensembl.org/Ovis_aries/Location/View?db=core;g=ENSOARG00000006407;r=6:80712077-81089105;t=ENSOART00000006970) | | 80712077 | | 81089105 | | |
| ERBB4 | Erb-b2 receptor tyrosine kinase 4 | | 2 | | 212081308 | | 212484401 | | |
| FARP1 | FERM ARH/RhoGEF and pleckstrin domain protein 1 | | [10](http://www.ensembl.org/ovis_aries/Location/View?r=10:74563400-74799926:1) | | 74563400 | | 74799926 | | |
| FGFR2 | fibroblast growth factor receptor 2 | | 22 | | 40407331 | | 40510126 | | |
| FHOD3 | Formin homology 2 domain containing 3 | | 23 | | 20610958 | | 21057595 | | |
| FZD3 | Frizzled class receptor 3 | | 2 | | 101761846 | | 101843767 | | |
| GRIK2 | Glutamate ionotropic receptor kainate type subunit 2 | | [8](http://www.ensembl.org/ovis_aries/Location/View?r=8:34947839-35493542:-1) | | 34947839 | | 35493542 | | |
| HCN4 | Hyperpolarization activated cyclic nucleotide gated potassium channel 4 | | [15](http://www.ensembl.org/homo_sapiens/Location/View?r=15:73319859-73369264:-1) | | 73319859 | | 73369264 | | |
| HEG1 | Heart development protein with EGF like domains 1 | | [1](http://www.ensembl.org/ovis_aries/Location/View?r=1:187691865-187930951:-1) | | 187691865 | | 187930951 | | |
| INADL | InaD-like | | [1](http://www.ensembl.org/Ovis_aries/Location/View?db=core;g=ENSOARG00000008998;r=1:36643319-37041253) | | 36643319 | | 37041253 | | |
| ISPD | Isoprenoid synthase domain containing | | [4](http://www.ensembl.org/ovis_aries/Location/View?r=4:24627906-25005090:-1) | | 24627906 | | 25005090 | | |
| LAMA2 | laminin alpha 2 | | 8 | | 54371404 | | 54895696 | | |
| LAMA3 | laminin alpha 3 | | [23](http://www.ensembl.org/ovis_aries/Location/View?r=23:32998185-33254634:-1) | | 32998185 | | 33254634 | | |
| MATN2 | matrilin 2 | | 9 | | 78779530 | | 78931679 | | |
| MEF2A | myocyte enhancer factor 2A | | 18 | | 5979082 | | 6166480 | | |
| MPP5 | Membrane palmitoylated protein 5 | | [7](http://www.ensembl.org/ovis_aries/Location/View?r=7:76040423-76113874:1) | | 76040423 | | 76113874 | | |
| MYCBP2 | MYC binding protein 2 E3 ubiquitin protein ligase | | [10](http://www.ensembl.org/ovis_aries/Location/View?r=10:52632844-52906106:-1) | | 52632844 | | 52906106 | | |
| MYH11 | myosin heavy chain 11 smooth muscle | | 24 | | 14094951 | | 14204211 | | |
| MYO18B | myosin XVIIIB | | 17 | | 65228017 | | 65452813 | | |
| NEDD4L | Neural precursor cell expressed developmentally down-regulated 4-like E3 ubiquitin protein ligase | | [23](http://www.ensembl.org/ovis_aries/Location/View?r=23:57413655-57753407:1) | | 57413655 | | 57753407 | | |
| NFASC | neurofascin | | 12 | | 1935014 | | 2090555 | | |
| NRCAM | neuronal cell adhesion molecule | | 4 | | 49251511 | | 49489760 | | |
| NRXN2 | neurexin 2 | | 21 | | 42166483 | | 42266002 | | |
| NRXN3 | neurexin 3 | | 7 | | 86760256 | | 87154967 | | |
| NTN1 | netrin 1 | | 11 | | 28031344 | | 28218243 | | |
| PDZRN3 | PDZ domain containing ring finger 3 | | [19](http://www.ensembl.org/ovis_aries/Location/View?r=19:28339040-28399126:1) | | 28339040 | | 28399126 | | |
| PRKCA | Protein kinase C alpha | | [11](http://www.ensembl.org/ovis_aries/Location/View?r=11:61554161-61834878:1) | | 61554161 | | 61834878 | | |
| SOX6 | SRY-box 6 | | 15 | | 35537489 | | 36100232 | | |
| SPTB | Spectrin beta erythrocytic | | [7](http://www.ensembl.org/ovis_aries/Location/View?r=7:73801139-73870135:-1) | | 73801139 | | 73870135 | | |
| TRPC7 | Transient receptor potential cation channel subfamily C member 7 | | [5](http://www.ensembl.org/ovis_aries/Location/View?r=5:45013059-45153419:-1) | | 45013059 | | 45153419 | | |
| TRPV4 | Transient receptor potential cation channel subfamily V member 4 | | [17](http://www.ensembl.org/ovis_aries/Location/View?r=17:63068250-63091201:1) | | 63068250 | | 63091201 | | |
| WNT7A | wingless-type MMTV integration site family member 7A | | 19 | | 57988969 | | 58046629 | | |
| ZFPM2 | zinc finger protein FOG family member 2 | | 9 | | 71461674 | | 71890059 | | |
| CGIs | | | | | | | | |  |
| GENE | | Description | | Chromosome | | start | | end |  |
| ACHE | | acetylcholinesterase | | 24 | | 35649479 | | 35653376 |  |
| CFAP46 | | Cilia and flagella associated protein 46 | | [22](http://www.ensembl.org/ovis_aries/Location/View?r=22:50211704-50500834:-1) | | 50211704 | | 50500834 |  |
| CHRNA4 | | cholinergic receptor nicotinic alpha 4 (neuronal) | | 13 | | 53570788 | | 53580288 |  |
| CHRNB2 | | cholinergic receptor nicotinic beta 2 (neuronal) | | 1 | | 103382196 | | 103390463 |  |
| CHRNB4 | | cholinergic receptor nicotinic beta 4 (neuronal) | | 18 | | 30045723 | | 30061504 |  |
| DNAAF5 | | dynein | | 24 | | 41627021 | | 41649086 |  |
| NAV1 | | neuron navigator 1 | | 12 | | 78762058 | | 78878846 |  |

**Supplementary Table S4.** Genes found to be significant enriched after pathway analysis for variation in methylation occurred in gene body GENEs (DMRs n=506 FDR<10exp-7) and CpG island CGIs (DMRs n=398 FDR<0.05). For each gene, chromosome location and description were reported.
