# Supplementary material for: Genome-Wide Epigenetic Characterization of Tissues from Three Germ Layers Isolated from Sheep Fetuses
Source: Front Genet. 2017 Sep 4;8:115. doi: 10.3389/fgene.2017.00115 (PMC5591608; doi:10.3389/fgene.2017.00115)
Supplement: Supplementary file 5 [file Data_Sheet_1.DOCX]

**Supplementary Figure S1.** Venn diagram of hypo-methylated regions (DNA methylation ≤ 20%) and hyper-methylated regions (DNA methylation ≥ 80%) found in three tissues (EC, M and EN) in CGIs.
