# Supplementary material for: Genome-Wide Epigenetic Characterization of Tissues from Three Germ Layers Isolated from Sheep Fetuses
Source: Front Genet. 2017 Sep 4;8:115. doi: 10.3389/fgene.2017.00115 (PMC5591608; doi:10.3389/fgene.2017.00115)
Supplement: Supplementary file 6 [file Data_Sheet_2.DOCX]

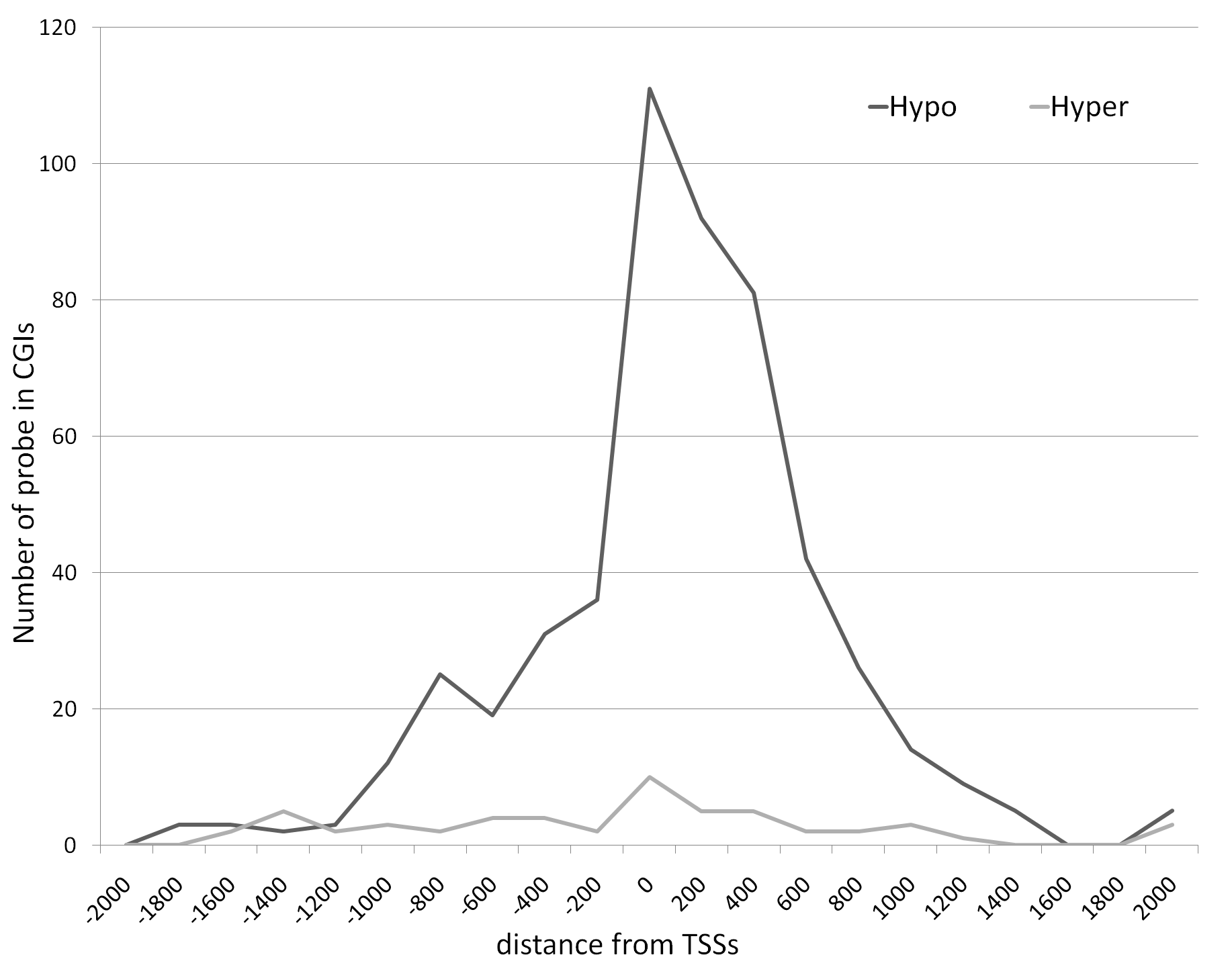


**Supplementary Figure S2.** Distribution of transcriptional start site TSSs, found in hypo-methylated and hyper-methylated CpG islands CGIs. On the x axis distances from TSSs were reported.
