# Supplementary material for: Genome-Wide Epigenetic Characterization of Tissues from Three Germ Layers Isolated from Sheep Fetuses
Source: Front Genet. 2017 Sep 4;8:115. doi: 10.3389/fgene.2017.00115 (PMC5591608; doi:10.3389/fgene.2017.00115)
Supplement: Supplementary file 7 [file Data_Sheet_3.DOCX]

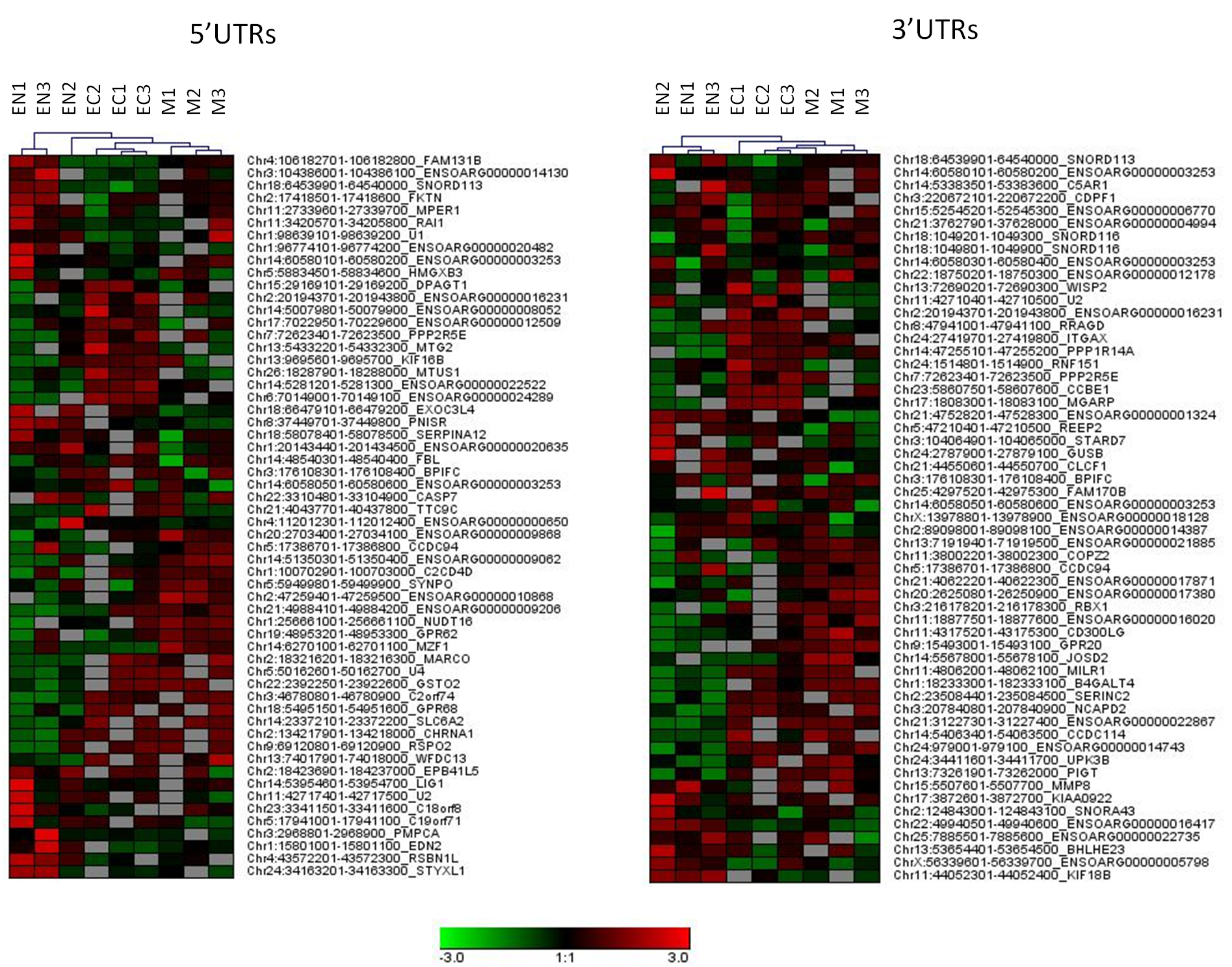


Figure 3. Hieralchical clustering for DMRs found in 5’UTR and 3’UTR. Each tissue was compared with other two tissues and 20 more hyper and 20 more hypo methylated DMRs from each comparison were used for clustering samples.
